# The Influence of the Grid Density of Measurement Points on Damage Detection in an Isotropic Plate by the Use of Elastic Waves and Laser Scanning Doppler Vibrometry

**DOI:** 10.3390/s21217394

**Published:** 2021-11-07

**Authors:** Łukasz Doliński, Marek Krawczuk, Magdalena Palacz, Wiktor Waszkowiak, Arkadiusz Żak

**Affiliations:** 1 Department of Biomechatronics, Faculty of Electrical and Control Engineering, Gdańsk University of Technology, Narutowicza 11/12, 80-233 Gdańsk, Poland; lukasz.dolinski@pg.edu.pl (Ł.D.); wiktor.waszkowiak@pg.edu.pl (W.W.); arkadiusz.zak@pg.edu.pl (A.Ż.); 2 Institute of Mechanics and Mechanical Engineering, Faculty of Mechanical Engineering and Ship Technology, Gdańsk University of Technology, Narutowicza 11/12, 80-233 Gdańsk, Poland; marek.krawczuk@pg.edu.pl; 3 Department of Production Engineering, Faculty of Organisation and Management, Silesian University of Technology, Akacemicka 2a, 44-100 Gliwice, Poland

**Keywords:** laser doppler scanning vibrometery, time-domain spectral finite element method, damage detection

## Abstract

Damage detection in structural components, especially in mechanical engineering, is an important element of engineering practice. There are many methods of damage detection, in which changes in various parameters caused by the presence of damage are analysed. Recently, methods based on the analysis of changes in dynamic parameters of structures, that is, frequencies or mode shapes of natural vibrations, as well as changes in propagating elastic waves, have been developed at the highest rate. Diagnostic methods based on the elastic wave propagation phenomenon are becoming more and more popular, therefore it is worth focusing on the improvement of the efficiency of these methods. Hence, a question arises about whether it is possible to shorten the required measurement time without affecting the sensitivity of the diagnostic method used. This paper discusses the results of research carried out by the authors in this regard both numerically and experimentally. The numerical analysis has been carried out by the use of the Time-domain Spectral Finite Element Method (TD-SFEM), whereas the experimental part has been based on the measurement performed by 1-D Laser Doppler Scanning Vibrometery (LDSV).

## 1. Introduction

The inspection of the technical condition of machinery and mechanical components has always been a fundamental part of engineering practice. The answer to the question about the technical condition of the equipment in service is crucial, among other things, for the reasons of operational safety and economic calculations. In recent years, various methods of the monitoring of engineering structures have been developed. A system that involves observation of the condition of a structure by means of control and measurement in order to detect, locate, identify and predict the development of deformation and damage, which may cause the structure to fail, is called SHM (Structural Health Monitoring) [1,2]. SHM is based on sensing to monitor the behaviour of structures, assess their performance, and identify damage at its early stage [3,4,5,6,7,8].

Generally, damage detection techniques can be divided into active and passive approaches [9,10]. The active approaches need an external excitation of the monitored structures, while their responses are measured using sensors. The passive approaches depend on sensor measurements for the detection of unknown inputs, such as external loads or impacts, environmental factors, and new damage, which causes changes in sensor measurements. The active approaches require sophisticated equipment and less signal processing to detect damage, whereas the passive approaches need more signal processing and less advanced equipment in most of the cases [11].

Non-destructive damage detection techniques can also be classified as local and global. If the potential damage location is already known for testing, then it is called a local damage detection technique. However, in some large and complex structures with inaccessible regions, it is not possible to locate damage using local damage detection techniques. In such cases, global damage detection techniques are necessary.

Vibration-based damage detection techniques are an example of global damage detection techniques [12,13,14,15,16,17,18,19,20,21]. Among the many available dynamical parameters, the propagation of elastic waves appears to be the most desirable, especially for damage in the earliest state of its growth. Gathering necessary information from signals in the form of propagating waves may be performed numerically and experimentally. In both these cases, sufficient accuracy is required to provide the carrier information for the respective processing algorithms.

As the measurement accuracy is usually limited by hardware capabilities, the selection of a suitable measuring tool needs to be optimised in terms of costs and required precision. When it comes to recording mechanical wave propagation signals, however, the class of instrumentation is essential. An excellent piece of equipment for registering propagating waves is a Laser Scanning Doppler Vibrometer (LSDV). The main advantage of such a system is measurement automation, high sensitivity and the non-contact nature of measurements. LSDV allows entire surfaces to be scanned quickly and accurately by using flexible and interactively created grids of measurement points.

On the other hand, the numerical sensitivity is limited to the proper mathematical model used for solutions. Various approaches to the proper modelling of complicated wave propagation phenomena can be found in the literature. For example, analytical models for modelling wave propagation in plate elements made out of various composite materials have been proposed in [22,23,24]. Numerical or grid-based methods [25,26,27] have found a wide range of applications to solve elastic wave propagation problems arising in seismology, medical ultrasound and non-destructive evaluation [28,29] or even textile industry [30]. However, the most versatile method for modelling wave propagation has been the Time-domain Spectral Finite Element Method (TD-SFEM) [31]. The main advantage of TD-SFEM is a more precise representation of high frequency signals than in the case of the classical Finite Element Method (FEM). This is especially important in the context of numerical calculations related to modelling wave propagation. Moreover, it is well known that thanks to the orthogonality of high-order approximation polynomials used in TD-SFEM, the diagonal forms of the inertia matrix are obtained. This additionally improves the numerical performance of the method.

Properly selected methods of dynamic parameter modelling used in damage detection algorithms allow for a various analysis to be carried out with the purpose of optimising these diagnostic methods. This process can cover many aspects, like: assessment of the FE model sensitivity [32]; analysis of the measurement signal in order to locate damage [33]; assessment of the effectiveness of a damage detection technique in terms of the density of the measurement grid [34,35,36,37,38] and the analysis of the influence of the location of the sensor grid on the proposed methodology correctness [39,40,41].

For both experimental and computational methods, special attention should be paid to the density of the grid of measurement points. The more the measuring points, the more accurate the representation of the measured quantity. On the other hand, the measurement time, dependent on the mesh density as well, is an important issue in industrial applications, since taking objects out of service can result in unavoidable financial costs. In the case of numerical investigations performed for complex geometries, there might be a difficulty resulting from the computational power of the equipment available. However, in the case of hardware measurements, the issue is very important, as the reduction of the number of measurement points allows for a significant reduction of measurement time, which in industrial conditions is of fundamental importance.

Based on the literature review carried out, it can be concluded that within the references found, there are no studies that clearly address the problem of the analysis of the influence of the grid density, both measured and calculated, on the sensitivity of the diagnostic method. Therefore, the current study aims to address this, both numerically and experimentally, in the case of an epoxy-glass plate containing delamination as well as for various grid densities of spectral finite elements/measurement points. In the analysis the Root Mean Square (RMS) values of wave signals have been chosen for the damage location. This is due to the fact that guided elastic waves reflecting from damage change the distribution of the energy, influencing such signals. These energy changes are then represented as variations in the values of RMS and can be successfully used for damage detection [33,42,43].

## 2. The Problem Analysed

**Figure 1:**
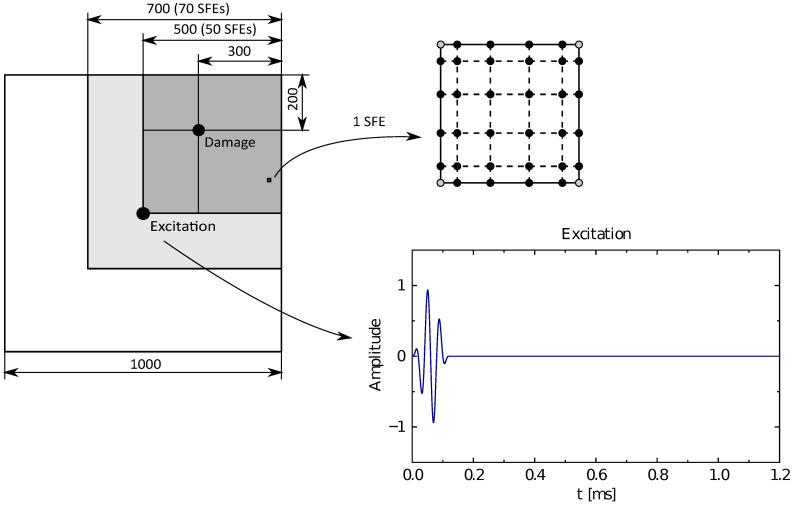
Examined composite panel with 1 SFE, nodes distribution and excitation signal.

Numerical and experimental investigations discussed have been carried out on a selected panel made out of plexiglass (Young’s modulus of 2.7 GPa, Poisson’s ratio equal to 0.31, density assumed as 1085 kg/m${}^{3}$). The geometrical dimensions of the analysed panel are shown in Figure 1. As the damage a small (11 mm diameter) plexiglass disc glued to the panel surface was considered (0.037% of the total panel mass). In the numerical model as damage, an equivalent additional mass has been considered, added to selected nodes of spectral finite elements (SFEs).

For numerical modelling, TD-SFEM has been chosen [31]. TD-SFEM is a computational technique that combines the properties of the polynomial approximation of spectral methods and the approach to the discretisation of the analysed area inherent in the finite element method (FEM) [44]. The property of FEM is that for each simple geometric object, specific points (called nodes) with certain approximating functions (called shape functions or node functions) are determined. These functions describe the distribution of the analysed physical properties inside finite elements (FEs) and at its boundaries. A characteristic of TD-SFEM is a non-uniform distribution of nodes within SFEs, which results from the distance between the roots of certain polynomials. This helps to avoid the Runge phenomenon [45], that is, large oscillations of approximating polynomials near the edges FEs. Therefore, high-order polynomials are used in TD-SFEM, which is impossible in the case of uniformly distributed nodes in the classical FEM [46].

The analysed panel has been modelled by six-node shell SFEs derived using TD-SFEM, described in more detail here [44]. The node distribution for the examined SFE has been based on Lobatto polynomials [47] and is presented in Figure 1b. In order to analyse the panel, 50 × 50 SFEs have been employed with the final grid size of 250 × 250 nodes. Additionally, an absorbing layer consisting of 2400 SEFs [48] has been assumed to conduit propagating waves out of the system. The panel has been excited with a sine wave signal (three modulations) modulated by a Hanning window [49] with a carrier frequency of 25 kHz. The total measurement time has been assumed as 1.2 ms, whereas the results have been recorded at the same moment in each case, that is at 0.8 ms. An illustration of the geometrical dimensions of the analysed panel is shown in Figure 1. This figure also shows the waveform of the excitation signal, as well as the distribution of nodes in one SFE. Additionally, it is worth mentioning that the position of SFE corner nodes remains the same as the position of the measurement points in LDSV.

**Figure 2:**
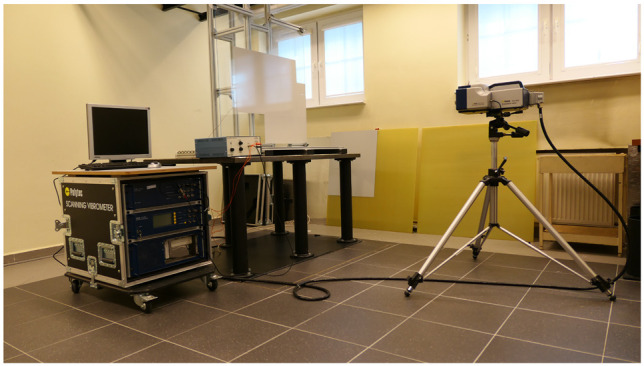
Experimental setup.

The experimental part of measurements has been carried out with LDSV Polytec PSV-400 [50] (a picture of the measuring system is shown in Figure 2). The most important part of this measuring system is the scanning head with a laser Doppler vibrometer, which allows fast, accurate, and non-contact measurements of vibrations of the whole surface under investigation. The main advantages of such a sensor are: measurement automation, high sensitivity and the non-contact nature of measurements. This approach allows entire surfaces to be scanned quickly and accurately by using flexible and interactively created grids of measurement points. LDSV automatically moves to each point on the scanning grid points, measures responses, and validates the measurements by checking the signal-to-noise ratio. In order to ensure the optimal measurement conditions, high scattering of the laser beam reflected from the measurement surface should be avoided because even a small amount of light returning to the sensor head can cause measurement interference. To compensate for this effect, the measured area should be covered with a reflective foil. The characteristic feature of such a foil is that it reflects light in the direction of its source. The full wave-field measurements of out-of-plane velocities were carried out on the surface opposite to a piezoelectric transducer. All measurements were performed in equally spaced grids with varying numbers of points, covering the whole surface of the specimen. Ten times averaging for each recorded time response was used to obtain higher signal-to-noise ratio.

## 3. Results

The results presented, both numerical and experimental, have been carried out for a successively reduced number of measurement points. First, the maximum possible grid of points (i.e., 250 × 250 in *x* and *y* direction, respectively) was analysed. All registered time signals have been used to determine the Root Mean Squared (RMS) value for each point as:

(1) $$RMS=\sqrt{\frac{\sum_{n=1}^{N}f_{n}^{2}}{N}},$$

where $f_{n}^{2}$ is a squared vibration magnitude (velocities) of a sample *n*, and *N* is the total number of samples used for RMS calculation. Due to the fact that elastic waves have been reflected from damage, the distribution of energy has also been changed. These energy changes have been represented as the maximal values of RMS and that fact has been used for damage location. Therefore, a map of RMS values, calculated for the simulated and measured signals, were chosen to visualise the location of possible damage. Moreover, in the analysed cases, the location of damage has been known since the authors wanted to verify if it is possible to locate the damage of a known position by a reduced number of measurement points.

**Figure 3:**
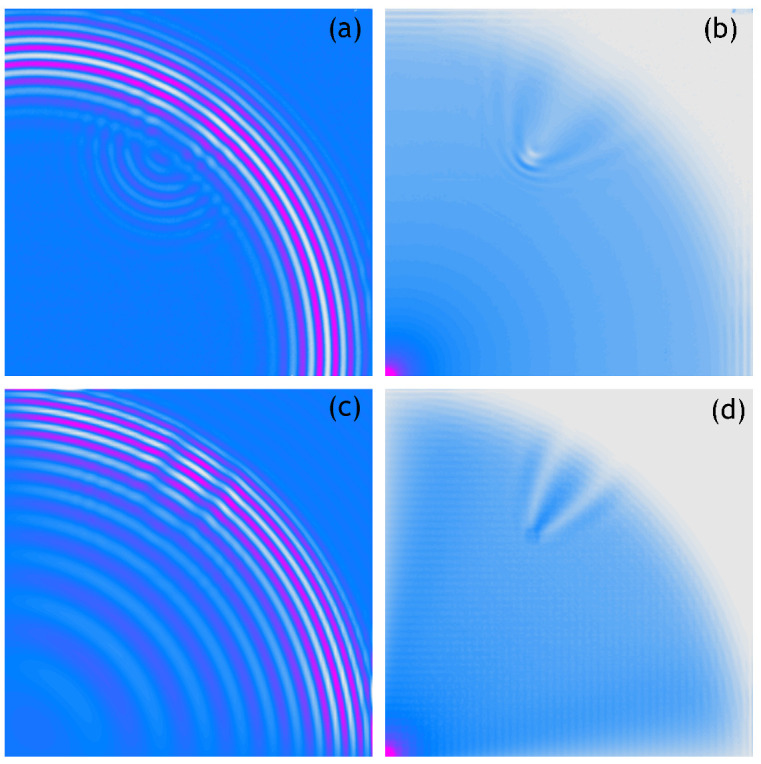
Wave propagation velocity patterns registered at 0.8 ms with 250 × 250 mesh grid, (**a**) Experimental data, (**b**) RMS of experimental data, (**c**) Calculated data, (**d**) RMS of calculated data.

The obtained results are shown in Figure 3. The figure presents four graphs. The top two—Figure 3a,b—refer to the results obtained experimentally, while the bottom two—Figure 3c,d—refer to the numerical analysis. The graph marked (a) shows a velocity pattern measured at the selected time instance, that is, 0.8 ms. The graph marked with (b) shows an RMS pattern based on each of the measured velocities from plot (a). The bottom two graphs show the results of numerical simulations recorded at the same time instances as the measurements. Similarly to the upper graph, the graph marked with (c) is a recorded velocity pattern and the graph marked with (d) is an RMS pattern based on it. From the graphs presented, it can be concluded that the maximal of the analysed number of measurement points allows for a perfect representation of the phenomenon under consideration.

**Figure 4:**
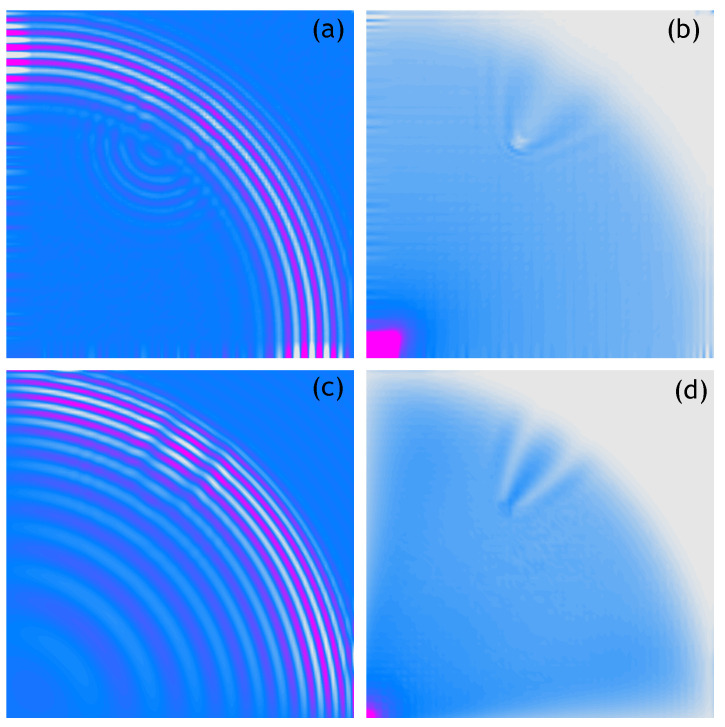
Wave propagation velocity patterns registered at 0.8 ms with 100 × 100 mesh grid, (**a**) Experimental data, (**b**) RMS of experimental data, (**c**) Calculated data, (**d**) RMS of calculated data.

**Figure 5:**
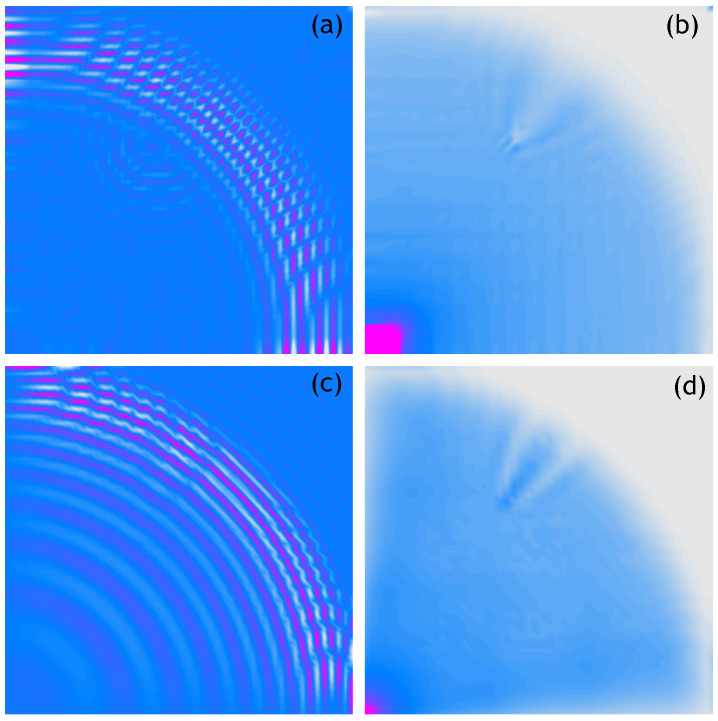
Wave propagation velocity patterns registered at 0.8 ms with 50 × 50 mesh grid, (**a**) Experimental data, (**b**) RMS of experimental data, (**c**) Calculated data, (**d**) RMS of calculated data.

**Figure 6:**
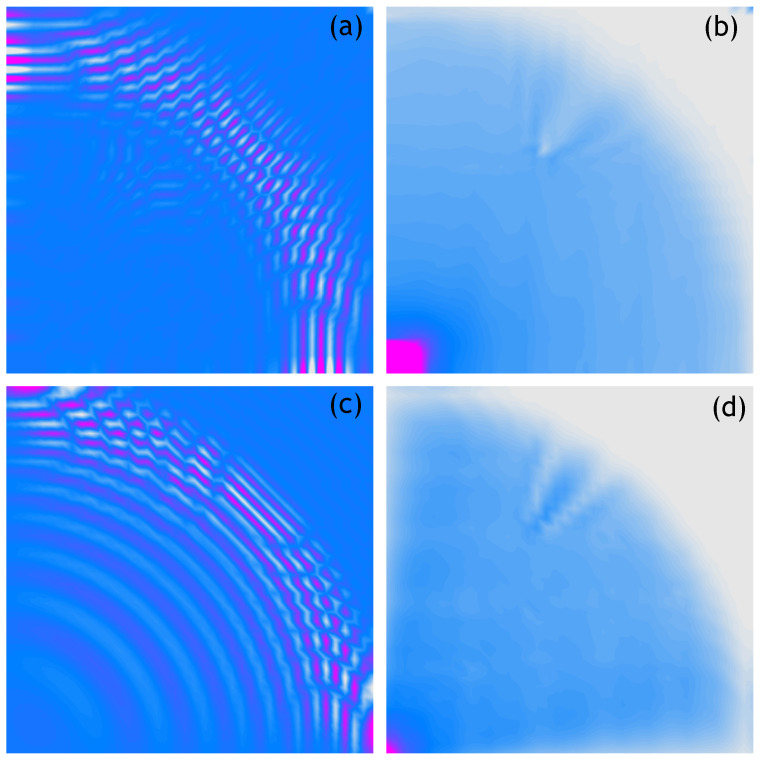
Wave propagation velocity patterns registered at 0.8 ms with 40 × 40 mesh grid, (**a**) Experimental data, (**b**) RMS of experimental data, (**c**) Calculated data, (**d**) RMS of calculated data.

**Figure 7:**
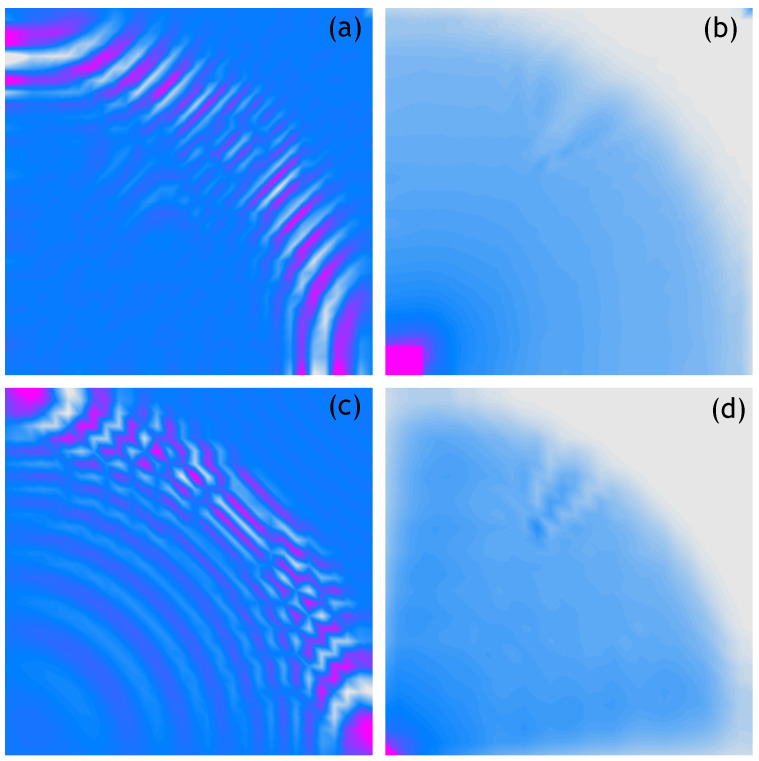
Wave propagation velocity patterns registered at 0.8 ms with 30 × 30 mesh grid, (**a**) Experimental data, (**b**) RMS of experimental data, (**c**) Calculated data, (**d**) RMS of calculated data.

The following figures show analogous results of measurements and calculations obtained for respectively sparser measurement grids. Thus, Figure 4 shows the 100 × 100 measurement points/nodes grid, Figure 5 presents the 50 × 50 measurement points/nodes grid, Figure 6 the 40 × 40 measurement points/nodes grid and finally Figure 7 the 30 × 30 measurement points/nodes grid. Based on the analysis of the results presented in these graphs it can be concluded that it is possible to identify the position of the defect for a grid even five times more sparse, that is, 50 × 50 measuring points in the *x* and *y* axis directions.

In order to determine the threshold grid size, for which damage localisation is possible, the analysis was carried out, the results of which are summarised in Table 1. For each of the signals recorded either experimentally or by numerical RMS has been calculated. Then, the damage location was determined (a simple mathematical procedure consisting of determination the coordinates for which the RMS derivative has been equal to zero). From the data in Table 1 it can be concluded that it has been possible to locate the damage for a grid consisting of only 40 × 40 measurement points. A smaller number of measurement points made it not possible to locate the damage.

A coherent summary of the results of the conducted studies can be found in Table 2. This table summarises information on the total number of measurement points, the percentage reduction in size of each of the grids analysed (% grid${}_{max}$), the ratio of the damage area to the area of one FE (A${}_{dam}$/A${}_{el.mesh}$), the duration time of the measurements (time [h]) and the results of the damage location identification for each case analysed. From the data given in the Table 2, it can be concluded that reducing the grid density even to less than 10% of grid${}_{max}$, in relation to the base grid density, allows the location of damage to be correctly estimated. If the grid is even more sparse, this can lead to an incorrect estimation of the damage position. It is also worth noting the profit in terms of calculation and measurement time. It can be clearly seen that it is possible to reduce the measurement time for this panel by LSDV below 1 h. This time saving is satisfactory as it allows us to significantly shorten the necessary time in many SHM applications.

## 4. Discussion and Conclusions

The main conclusion that arises from the investigations carried out is that propagating elastic waves represents a very sensitive and precise tool, which allows for the identification of even small defects in structural elements, both based on numerical data, as well as recorded from experimental measurements. However, in the case of wave propagation analysis, it should be kept in mind that the effectiveness of such a diagnostic approach largely depends on the signal processing algorithm that is implemented. In the current example, the RMS of wave signal velocities has been chosen for that purpose. It is due to the fact that guided elastic waves reflect from damage, which changes the distribution of the energy of such signals. These energy changes are represented as variations in the values of RMS and can be successfully used for damage detection. The proposed approach to fault detection problems is known in the literature (e.g., in [33]). However, it is worth noting that the results related to the analysis of the influence of the density of the grids of measurement points on the sensitivity and accuracy of the discussed approach remains unknown in the literature.

The most important conclusion from the completed analysis is that it is possible to correctly locate the position of damage even in the case of a reduced measurement grid. This is possible, provided that a diagnostic method exploiting the phenomenon of elastic wave propagation and the determination of the RMS of measured signals are employed. The reduction of the grid density should not exceed 10% of the total number of measurement points of the output grid (below 10% of grid${}_{max}$, in relation to the base grid density). Such a recommendation and similar ones apply to both grids used for numerical calculations and grids used for experimental measurements.

The information about the correct identification of the defect position, even in the case of the reduced grid density, is essential from the point of view of calculation and measurement times. This considerably reduces the time necessary to take an object out of service, and this is directly related to the financial side of the diagnostic process. For example, as seen from Table 2, it is possible to significantly shorten the measurement time from 8 h to about 40 min without compromising the correctness of the fault location. The authors believe that this is a very promising result, which may be of interest to those who are involved in the technical side of the application of increasingly common diagnostic methods based on the elastic wave propagation phenomenon.

The primary reason for the differences observed between the experimental and simulation results comes from damping. In the experiment, high frequency signal components propagating at higher velocities (greater wavelengths) are more attenuated. In the numerical simulation this is not observed due to no damping being assumed in the model. Additionally, it is worth mentioning that the authors’ intention was to investigate the possibility of reducing the measurement grid density in terms of the effectiveness of the damage location assessment method. The described approach is of a research character; it is a suggestion of a change to the approach to experimental and numerical investigations. Usually, in such cases, tests are carried out on basic objects in laboratory conditions. The authors plan to extend their research by verifying the influence of additional operating conditions, such as signal noise or damping in the model.

Finally, it is worth highlighting the most important conclusions from the research results presented in this work and discussed:

- The reduction of the measurement grid density, in numerical and experimental analyses, allows one to locate the damage correctly using the proposed damage detection technique;
- The reduction of the same grid density does not affect the precision of the localisation process;
- The use of a reduced grid significantly also allows one to reduce the measurement time in a significant manner without compromising its sensitivity (Table 2).

## Figures and Tables

**Table 1:** Damage location (in cm from plate edges) identified by the use of RMS of calculated and measured velocities.

Grid	Finite Element Size	Found x	Found y	Found x	Found y
	**[cm]**	**Simulation**		**Experiment**	
250	0.20	0.2048	0.3072	0.1996	0.3060
200	0.25	0.2010	0.3040	0.2060	0.3065
150	0.33	0.1980	0.2953	0.2047	0.3087
140	0.36	0.2014	0.3058	0.2050	0.3058
130	0.38	0.2016	0.3023	0.2093	0.3101
120	0.42	0.2059	0.3109	0.2017	0.3109
110	0.45	0.1927	0.2936	0.2064	0.3073
100	0.50	0.2020	0.2929	0.2071	0.3081
90	0.56	0.2022	0.3034	0.2022	0.3090
80	0.63	0.2025	0.3101	0.2025	0.3101
70	0.71	0.1957	0.3043	0.2029	0.3116
60	0.83	0.1949	0.2966	0.2034	0.3136
50	1.00	0.1939	0.2959	0.2041	0.3163
40	1.25	0.2051	0.2949	0.1923	0.2949
30	1.67	0.2241	0.3276	general region	
20	2.50	general region		general region	
10	5.00	not possible		not possible	

**Table 2:** Summary of damage localisation analysis in terms of number of measurement points, percentage measurement grid reduction and the change of damage area.

Grid ID	No. of Points	% Grid${}_{\max}$	A${}_{\mathrm{dam}}$/A${}_{\mathrm{el.mesh}}$	Time [h]	RMS	Signal
250	62,500	100.00	0.78540	≈8	+	+
200	40,000	64.00	0.50265	5.1200	+	+
150	22,500	36.00	0.28274	2.8800	+	+
140	19,600	31.36	0.24630	2.5088	+	+
130	16,900	27.04	0.21237	2.1632	+	+
120	14,400	23.04	0.18096	1.8432	+	+
110	12,100	19.36	0.15205	1.5488	+	+
100	10,000	16.00	0.12566	1.2800	+	+
90	8100	12.96	0.10179	1.0368	+	+
80	6400	10.24	0.08042	0.8192	+	+
70	4900	7.84	0.06158	0.6272	+	region
60	3600	5.76	0.04524	0.4608	+	region
50	2500	4.00	0.03142	0.3200	+	-
40	1600	2.56	0.02011	0.2048	region	-
30	900	1.44	0.01131	0.1152	region	-
20	400	0.64	0.00503	0.0512	region	-
10	100	0.16	0.00126	0.0128	-	-

## Abbreviations

The following abbreviations are used in this manuscript:

LSDV	Laser Scanning Doppler Vibrometer
SFEM	Spectral Finite Element Method
RMS	Root Mean Square

## References

[1] Hamdan A., Sultan M.T.H., Mustapha F., Jawaid M., Thariq M., Saba N. (2019). Structural health monitoring of biocomposites, fibre-reinforced composites, and hybrid composite. Structural Health Monitoring of Biocomposites, Fibre-Reinforced Composites and Hybrid Composites.

[2] Stawiarski A., Muc A. (2019). On Transducers Localization in Damage Detection by Wave Propagation Method. Sensors.

[3] Lee J. (2000). Free vibration analysis of delaminated composite beams. Comput. Struct..

[4] Takeda S., Okabe Y., Takeda N. (2002). Delamination detection in CFRP laminates with embedded small-diameter fiber Bragg grating sensors. Compos. Part Appl. Sci. Manuf..

[5] Zou Y., Tong L., Steven G. (2000). Vibration-based model-dependent damage (delamination) identification and health monitoring for composite structures—A review. J. Sound Vib..

[6] Mook G., Pohl J., Michel F., Benziger T. Non-destructive Inspection of Smart Materials. Proceedings of the 15th World Conference on Nondestructive Testing.

[7] Todorovska M.I., Rahmani M.T.T. (2013). System identification of buildings by wave travel time analysis and layered shear beam models—Spatial resolution and accuracy. Struct. Control. Health Monit..

[8] Skłodowska A.M., Holden C., Guéguen P., Finnegan J., Sidwell G. (2021). Structural change detection applying long-term seismic interferometry by deconvolution method to a modern civil engineering structure (New Zealand). Bull. Earthq. Eng..

[9] Senthilkumar M., Sreekanth T., Manikanta Reddy S. (2021). Nondestructive health monitoring techniques for composite materials: A review. Polym. Polym. Compos..

[10] Staszewski W.J., Mahzan S., Traynor R. (2009). Health monitoring of aerospace composite structures—Active and passive approach. Compos. Sci. Technol..

[11] Staszewski W.J., Boller C., Tomlinson G.R. (2003). Aircraft structural health and usage monitoring. Health Monitoring of Aerospace Structures: Smart Sensor Technologies and Signal Processing.

[12] Yan Y.J., Cheng L., Wu Z.Y. (2007). Development in vibration-based structural damage detection technique. Mech. Syst. Signal Process..

[13] Doebling S.W., Farrar C.R., Prime M.B. (1996). Damage Identification and Health Monitoring of Structural and Mechanical Systems from Changes in Their Vibration Characteristics: A Literature Review.

[14] Frigui F., Faye J.P., Martin C. (2018). Global methodology for damage detection and localization in civil engineering structures. Eng. Struct..

[15] Michel C., Gueguen P. (2018). Interpretation of the velocity measured in buildings by seismic interferometry based on Timoshenko beam theory under weak and moderate motion. Soil Dyn. Earthq. Eng..

[16] Hilloulin B., Zhang Y., Abraham O., Loukili A., Grondin F., Durand O., Tournat V. (2014). Small crack detection in cementitious materials using nonlinear coda wave modulation. NDT E Int..

[17] Obermann A., Planes T., Larose E., Sens-Schönfelder C., Campillo M. (2013). Depth sensitivity of seismic coda waves to velocity perturbations in an elastic heterogeneous medium. Geophys. J. Int..

[18] Planès T., Larose E., Margerin L., Rossetto V., Sens-Schöenfelder C. (2014). Decorrelation and phase-shift of coda waves induced by local changes: Multiple scattering approach and numerical validation. Waves Random Complex Media.

[19] Farrar C.R., Worden K. (2007). An introduction to structural health monitoring. Philos. Trans. R. Soc. Math. Phys. Eng. Sci..

[20] Limongelli M.P. (2019). SHM in some European countries. Seismic Structural Health Monitoring.

[21] Picozzi M., Parolai S., Mucciarelli M., Milkereit C., Bindi D., Ditommaso R., Zschau J. (2011). Interferometric analysis of strong ground motion for structural health monitoring: The example of the L’Aquila, Italy, seismic sequence of 2009. Bull. Seismol. Soc. Am..

[22] Barker L.M. (1971). A Model for Stress Wave Propagation in Composite Materials. J. Compos. Mater..

[23] Noiret D., Roget J. (1989). Calculation of Wave Propagation in Composite Materials Using the LAMB Wave Concept. J. Compos. Mater..

[24] Muc A., Stawiarski A.T. (2012). Wave propagation in composite multilayered structures with delaminations. Mech. Compos. Mater..

[25] Bathe K.J. (1996). Finite Element Procedures.

[26] Hughes T., Hulbert G. (1988). Space-time finite element methods for elasto-dynamics: Formulations and error estimates. Comput. Methods Appl. Mech..

[27] Ihlenburg F., Babuška I. (1995). Finite element solution of the Helmholtz equation with high wave number part I: The h-version of the FEM. Comput. Math. Appl..

[28] Van Pamel A., Sha G., Rokhlin S.I., Lowe M.J.S. (2016). Finite-element modelling of elastic wave propagation and scattering within heterogeneous media. Proc. R. Soc. A.

[29] Seriani G., Oliveira S.P. (2020). Numerical modeling of mechanical wave propagation. Riv. Nuovo C.

[30] Thierry V., Brown L., Chronopoulos D. (2018). Multi-scale wave propagation modelling for two-dimensional periodic textile composites. Compos. Part B Eng..

[31] Patera A.T. (1984). A spectral element method for fluid dynamics: Laminar flow in a channel expansion. J. Comput. Phys..

[32] Razavi M., Hadidi A. (2020). Assessment of sensitivity-based FE model updating technique for damage detection in large space structures. Struct. Monit. Maint..

[33] Radzieński M., Doliński L., Krawczuk M., Żak A., Ostachowicz W. Application of RMS for damage detection by guided elastic waves. Proceedings of the 9th International Conference on Damage Assessment of Structures (DAMAS 2011).

[34] Porcu M.C., Patteri D.M., Melis S., Aymerich F. (2019). Effectiveness of the FRF curvature technique for structural health monitoring. Constr. Build. Mater..

[35] Xu Y.F., Zhu W.D., Smith S.A. (2017). Non-model-based damage identification of plates using principal, mean and Gaussian curvature mode shapes. J. Sound Vib..

[36] Laflamme S., Cao L., Chatzi E., Ubertini F. (2017). Damage Detection and Localization from Dense Network of Strain Sensors. Shock Vib..

[37] Mathews V.J. (2014). Damage Mapping in Structural Health Monitoring Using a Multi-Grid Architecture. Proceedings of the 41st Annual Review of Progress in Quantitative Nondestructive Evaluation (QNDE).

[38] Chen H.L., Liu Z.H., Gong Y., Wu B., He C.F. (2021). Evolutionary Strategy-Based Location Algorithm for High-Resolution Lamb Wave Defect Detection With Sparse Array. IEEE Trans. Ultrason. Ferroelectr. Freq. Control.

[39] Uribe-Riestra G.C., Ocampo-Bello J.A., Gamboa F., Mendoza-Santoyo F., Perez-Lopez C., Franco-Urquiza E.A., Preud’homme M., Castillo-Atoche A., Aviles F. (2020). Influence of electrode configuration on impact damage evaluation of self-sensing hierarchical composites. J. Intell. Mater. Syst. Struct..

[40] Matveenko V., Kosheleva N., Serovaev G. (2019). Damage detection algorithm based on using surface mounted fiber-optic sensors on Bragg gratings. Procedia Struct. Integr..

[41] Ikikardaslar K.T., Delale F. (2018). Self-sensing damage in CNT infused epoxy panels with and without glass-fibre reinforcement. Strain.

[42] Żak A., Radzieński M., Ostachowicz W., Krawczuk M. (2012). Damage detection strategies based on propagation of guided elastic waves. Smart Mater. Struct..

[43] Sikdar S., Kudela P., Radzieński M. (2018). Kundu, A.; Ostachowicz, W. Online detection of barely visible low-speed impact damage in 3D-core sandwich composite structure. Compos. Struct..

[44] Ostachowicz W., Kudela P., Krawczuk M., Żak A. (2012). Guided Waves in Structures for SHM: The Time-Domain Spectral Element Method.

[45] Runge C.T. (1901). Über empirische Funktionen und die Interpolation zwischen äquidistanten Ordinaten. Z. Math. Phys..

[46] Palacz M. (2018). Spectral Methods for Modelling of Wave Propagation in Structures in Terms of Damage Detection—A Review. Appl. Sci..

[47] Hunter D., Nikolov G. (2000). On the error term of symmetric Gauss-Lobatto quadrature formulae for analytic functions. Math. Comput..

[48] Żak A., Krawczuk M., Skarbek Ł., Palacz M. (2014). Numerical analysis of elastic wave propagation in unbounded structures. Finite Elem. Anal. Des..

[49] Harris F.J.T. (1978). On the use of windows for harmonic analysis with the discrete Fourier transform. Proc. IEEE.

[50] (2021). PSV-400 User Manual. http://www.dbkes.com.tr/brosur/psv_400.pdf.

